# Age-related changes in mobility assessments correlate with repetitive goal-directed arm-movement performance

**DOI:** 10.1186/s12877-023-04150-3

**Published:** 2023-08-11

**Authors:** Isabelle Daniela Walz, Sarah Waibel, Andreas Kuhner, Albert Gollhofer, Christoph Maurer

**Affiliations:** 1 Department of Neurology and Neuroscience, Medical Center, Faculty of Medicine, University of Freiburg, Freiburg, Germany; 2 Department of Sports and Sport Science, University of Freiburg, Freiburg, Germany; 3 Department of Computer Science, University of Freiburg, Freiburg, Germany; 4 Present Address: Franka Emika GmbH, Freiburg, Germany

**Keywords:** Instrumented Timed Up and Go test, Gait speed, Dual-task, Fast gait speed, Fast repetitive targeted arm-movement

## Abstract

**Background:**

There is ample evidence that mobility abilities between healthy young and elderly people differ. However, we do not know whether these differences are based on different lower leg motor capacity or instead reveal a general motor condition that could be detected by monitoring upper-limb motor behavior. We therefore captured body movements during a standard mobility task, namely the Timed Up and Go test (TUG) with subjects following different instructions while performing a rapid, repetitive goal-directed arm-movement test (arm-movement test). We hypothesized that we would be able to predict gait-related parameters from arm motor behavior, even regardless of age.

**Methods:**

Sixty healthy individuals were assigned to three groups (young: mean 26 ± 3 years, middle-aged 48 ± 9, old 68 ± 7). They performed the arm-movement and TUG test under three conditions: preferred (at preferred movement speed), dual-task (while counting backwards), and fast (at fast movement speed). We recorded the number of contacts within 20 s and the TUG duration. We also extracted TUG walking sequences to analyze spatiotemporal gait parameters and evaluated the correlation between arm-movement and TUG results.

**Results:**

The TUG condition at preferred speed revealed differences in gait speed and step length only between young and old, while dual-task and fast execution increased performance differences significantly among all 3 groups. Our old group’s gait speed decreased the most doing the dual-task, while the young group’s gait speed increased the most during the fast condition. As in our TUG results, arm-movements were significant faster in young than in middle-aged and old. We observed significant correlations between arm movements and the fast TUG condition, and that the number of contacts closely predicts TUG time_fast_ and gait speed_fast_. This prediction is more accurate when including age.

**Conclusion:**

We found that the age-related decline in mobility performance that TUG reveals strongly depends on the test instruction: the dual-task and fast condition clearly strengthened group contrasts. Interestingly, a fast TUG performance was predictable by the performance in a fast repetitive goal-directed arm-movements test, even beyond the age effect. We assume that arm movements and the fast TUG condition reflect similarly reduced motor function.

**Trial registration:**

German Clinical Trials Register (DRKS) number: DRKS00016999, prospectively registered on March, 26, 2019.

## Introduction

There is ample research evidence that aging or specific diseases affect motor performance and can lead to functional impairments. Standardized clinical tests help to assess one’s mobility and to monitor its changes, e.g., for estimating the risk of falls. These tests often measure gait and balance capacity, since these are essential for autonomy in daily living activities and thus closely associated with our overall health and mortality [1]. Observation of these mobility tasks can be refined by motion capture systems providing objective and precise information about one’s motor behavior. Such assessments focus predominantly on lower limb performance, e.g., gait characteristics [2–5]. However, quantifying the motor behavior of individuals with considerably impaired mobility is challenging. Although there are specific upper-limb tests for assessing specific disease-related impairments [6–9], assessing upper-limb performance to gain a general impression of sensorimotor function is still uncommon.

Most studies applying kinematic measures for assessing upper-body performance focus on simple, single-joint movements, e.g., finger tapping or single reaching tasks [6, 7]. Our approach in the present study was therefore to introduce a repetitive multi-joint movement test for the upper limbs whose executive demands are more likely to resemble gait control mechanisms than those of less complex tasks. We aimed to compare the upper-limb performance with how subjects performed in a standard clinical mobility test.

For upper-limb performance analysis, we implemented a repetitive movement task challenging the sensorimotor system in terms of fast and goal-directed execution, i.e., an arm-movement test in sitting position. Disease- and age-related changes in upper-limb movements are generally quantifiable e.g., by slower movement speeds, reduced precision and smoothness, and greater variability [7, 10–13], and these may correlate with several health-related outcomes [10, 11, 14]. For example, there is evidence of an association between reduced arm-movement speed during a repetitive task and a higher mortality risk [15].

As a frequently applied mobility test acknowledged as clinically relevant, we chose the Timed Up and Go (TUG) test [16, 17]. The TUG test covers different demands of daily living: stand up and sit down, accelerated and decelerated walking (3 m), and turn around. The TUG test thus challenges especially neuromuscular and cognitive resources. As a proof-of-concept approach, we included healthy individuals of three age groups to evaluate age-related performance differences in TUG and arm-movement. Being aware of a ceiling effect in the TUG performance of well-conditioned individuals, we analyzed gait parameters during the TUG in addition to TUG time, and applied TUG under different conditions. To enable a differentiated perspective of sensorimotor functions, TUG is executed at the preferred movement speed as a reliable and approved sign of vitality [4, 18], while counting backwards induces cognitive-motor interference (dual-task) [3, 19], and done at a fast movement speed, it assesses acceleration abilities associated with disability and functional reserve capacity [20, 21]. To capture motion precisely, we applied an optoelectronic tracking system that calculates reliable whole-body position data.

The present study evaluates interrelations between upper- and lower-body performance. This may enable a deeper understanding of movement organization. We hypothesized that the upper-limb performance as measured via rapidly-executed, repetitive, goal-directed arm-movements (arm-movement test) would predict the (gait) performance in a functional highly-relevant mobility test such as TUG. We assumed that arm movements and TUG execution during varying conditions (preferred, dual-task, fast) would both enable us to quantify declining motor function even beyond a sole age effect.

## Materials and methods

### Participants

We enrolled a total of 60 healthy participants for three different age classes (young: n = 20, 18–34 years; middle-aged: n = 20, 35–59 years; old: n = 20, 60 + years). Our inclusion criteria were sufficient German language fluency and written informed consent. Exclusion criteria were: acute psychotic syndrome, medication or concomitant disease limiting the integrity of scientific data, concomitant disease and any additional disease impairing stance and gait or upper-limb function in a relevant way (e.g. painful arthrosis, recent joint replacement, peripheral neuropathy). All subjects underwent detailed anamnesis, including the number of falls within the last past half year as well as chronic disabilities requiring medical treatment. Furthermore, we clinically tested balance and mobility via the Performance Oriented Mobility Assessment (POMA) including 9 balance items (Score 0–16) and 8 gait items (Score 0–12), with a lower score indicating a high risk of falling [22]. We also assessed fear of falling via the Falls Efficacy Scale - International (FES-I) scaled from 16 to 64, with values < 20 indicating no, 20–27 a moderate, and 28–64 a high fear of falling [23]. Table 1 summarizes our subjects’ characteristics.

**Table 1:** Subjects’ characteristics

	Young (18–34 years) n = 20	Middle-aged (35–59 years) n = 20	Old (60 + years) n = 20	p-value
Age (years) mean ± SD (range)	26.4 ± 3.3 (19–32)	47.9 ± 9.0 (35–59)	67.8 ± 6.5 (61–81)	<.001 ^a, b, c^
Sex (m:f) n	11:9	10:10	11:9	0.935
Weight (kg) mean ± SD (range)	69.6 ± 13.6 (52–103)	76.1 ± 17.2 (50–100)	70.1 ± 13.7 (52–105)	0.314
Height (m) mean ± SD (range)	1.75 ± 0.9 (1.62–1.93)	1.73 ± 0.1 (1.58–1.89)	1.69 ± 0.1 (1.55–1.80)	0.098
BMI (kg/m²) mean ± SD (range)	22.8 ± 4.5 (17.2–38.2)	25.3 ± 4.6 (19.0–35.4)	24.5 ± 3.8 (19.4–33.9)	0.107
FES-I [scale 16–64] (range)	16.5 ± 0.6 (16–18)	17.4 ± 1.7 (16–21)	17.2 ± 1.5 (16–21)	0.294
POMA [scale 0–28] (range)	27.9 ± 0.3 (27–28)	27.7 ± 0.6 (26–28)	27.6 ± 0.6 (26–28)	0.151
Falls [past year] n (range)	0	0	3 (0–1)	0.045 ^a, b^
Chronic disabilities requiring medical treatment [n]				
- Coronary heart disease	0	1	2	
- Arterial hypertension	0	0	4	
- Diabetes mellitus	0	1	0	
- Celiac disease	1	0	0	
- Thyroid disease	1	3	2	
- Hepatitis B	0	0	1	
- Bowel disease	0	0	1	

SD, standard deviation; BMI, Body Mass Index; FES-I, Falls Efficacy Scale – International, values < 20 indicating no, 20–27 a moderate, 28–64 a high fear of falling; POMA, Performance Oriented Mobility Assessment, consisting of a balance and gait score with lower scores indicate a higher risk of falling

Bonferroni corrected post-hoc test reveals ^a^ significant difference between young and old aged, ^b^ significant difference between middle- and old aged, ^c^ significant difference between young and middle-aged

This study was approved by the Ethics Committee of the University of Freiburg and conducted according to the Declaration of Helsinki (German Register of Clinical Trials No.: DRKS00016999).

### Methods

All participants performed the Timed Up and Go (TUG) test in three different conditions twice: performing TUG first at preferred movement speed (preferred condition), then while executing an additional cognitive task (counting backwards in steps of two starting from 100 in the first and from 50 in the second trial [24]; dual-task condition), and finally as fast as possible without running, meaning one or both feet always in ground contact (fast condition). The instructions were standardized. Participants walked wearing their own footwear.

**Fig. 1:**
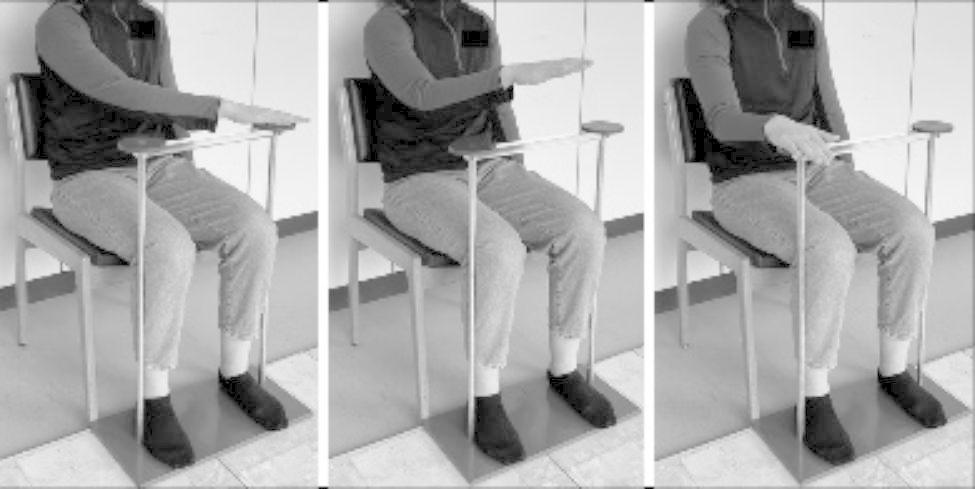
Repetitive goal-directed arm-movement

We also implemented a fast repetitive goal-directed arm-movement test to assess upper-limb movement in sitting position. Participants were instructed to touch two platforms alternating by one hand as fast as possible within a period of 20 s (arm-movement test) while sitting on a chair. The two platforms’ base is positioned on the floor in alignment with the participant’s feet position (Fig. 1). The platforms are 75 cm high, and the distance between them measures 26 cm. Each hand did the arm-movement test twice. This test originates from the water-pouring task in the Fahn‐Tolosa‐Marin Clinical Rating Scale for Tremor [25].

### Marker-less motion capture and data processing

All movement tasks were recorded via a marker-less vision-based motion capture system, i.e. TheCaptury (The Captury GmbH, Saarbrücken, Germany). This system provides reliable motion data [26, 27]. It uses a visual hull and a background subtraction method to estimate the subject’s silhouette. A skeleton is fitted into the subject by an automatic scaling process. The setup procedure involves calibrating each single camera (intrinsic calibration), the cameras to each other (extrinsic calibration) and the extraction of the background. These calibrations can be stored and used later, so the subject steps into the system and, after a short skeleton calibration (up to 60 s), the system is ready to record with 100 Hz at a resolution of 1 mm [28]. We recorded and analyzed data applying a live measurement program established by Kuhner et al. [27] that delivers TUG and arm-movement parameters immediately after each trial. We recorded the duration [s] needed to complete each TUG trial (TUG time).

To extract gait parameters from the TUG sequence, we divided it into four distinct segments: stand-up, turn, turn-to-sit, and walking. We took a sliding window approach to identify the transition points between these segments. Specifically, we sought a sequence of frames with a constant increase in hip-height to extract the starting point of standing-up, taking the inverse approach to find the endpoint of the stand-up phase. We determined the start and endpoints of the turn-to-sit segment similarly. The following method was used to detect the turning segment: first we identified the point farthest from the initial sitting position point, which served as the midpoint of the turning segment. Then we examined the frames before this midpoint to identify the point where the body started to rotate away from the walking direction. We analyzed the frames after the midpoint to pinpoint similarly - the point where the body completed the turn and resumed walking in the opposite direction. By cutting out the sit-to-stand, turn, and turn-to-sit movements, we isolated the walking segments, thus enabling us to calculate these gait parameters: step length [cm], average gait speed [cm/s], cadence [steps/min] and proportion of double support phase of a step [double support, %].

For the arm-movement test, we referred to the number of contacts per trial, maximum speed [m/s] achieved by one single arm-movement during one trial, and fatigue [%] over 20 s (percentage change in speed from the first to the last repetition) for analysis. We calculated the mean value of left and right hand for further data processing.

Each task, that is TUG conditions and arm-movement test, was performed twice and the mean value was calculated for data analysis as the intraclass correlation coefficient for the two trials ranging between 0.782 and 0.955, meaning substantial to almost perfect agreement.

### Data analysis

Pre-processed data were analyzed using Microsoft Excel version 16.0 (Microsoft Corporation, Redmond, Washington, USA) and IBM SPSS Statistics for Windows, version 26.0 (IBM Corp., Armonk, N.Y., USA). The illustrations were created with RStudio, version 4.0.3 (RStudio, PBC, Boston, USA). Participants’ characteristics were analyzed via the one-way ANOVA for weight and height; the Kruskall-Wallis test for BMI, FES-I, POMA, number of falls; and a chi-square test for gender.

Differences between groups were assessed by non-parametric analysis (Kruskal-Wallis ANOVA) as the assumption of normal distribution (Shapiro-Wilk test) was not satisfied for all parameters. Normal distribution was violated for TUG time_dual−task, fast_, velocity_normal, fast_, step length_normal, dual−task_, double-support_dual−task_, cadence_dual−task_, and fatigue (arm-movement test). To compare different TUG conditions, the Wilcoxon signed-rank test was used. For Wilcoxon signed-rank test and Post-Hoc results from Kruskal-Wallis ANOVA effect size r was calculated with r = z/√N, with r = 0.10 as a small effect, r = 0.30 a medium effect and r = 0.50 as a large effect size [29]. For Kruskal-Wallis ANOVA, eta-squared was calculated as effect size [30]. All results were presented with median and interquartile range (IQR). The level of statistical significance was set at *p* < 0.05. P-values of post-hoc comparisons were corrected by the Bonferroni’s procedure. Furthermore, we performed a correlation analysis (Pearson correlation test) between the two fast tasks, i.e. TUG variables at fast condition (_fast_) and arm-movement parameters. Based on our correlation results, we ran multiple regression analyses to predict TUG_fast_ performance based on arm-movement performance. These models include the two TUG_fast_ variables showing the strongest correlation to arm-movement as dependent variables, the parameters number of contacts and age as explanatory (independent) variables.

## Results

No adverse event occurred during the tests, and all participants performed all test conditions. We included data from N = 60 participants in our analysis. Participants’ characteristics revealed a group difference in the fall incidence with three reported falls in the old group, while groups did not differ in functional mobility (Tinetti POMA) or fear of falling (FES-I) (Table 1).

### Group differences in TUG conditions

#### Preferred condition

The time needed to complete the TUG test did not differ among groups. Furthermore, the parameters: double support time and cadence were similar among groups, while gait speed and step length revealed a significant group difference with a post-hoc difference between young and older adults (gait speed, r = 0.47, p = 0.010; step length, r = 0.41, p = 0.030). This shows that young participants walked faster by taking longer steps than the older ones (Table 2).

**Table 2:** Gait parameters of young, middle-aged, and older adults

Trial	Parameter	Young (18–34 years) median (IQR)	Middle-aged (35–59 years) median (IQR)	Old (60 + years) median (IQR)	p-value overall	η²
		n = 20	n = 20	n = 20	n = 60	n = 60
Preferred	TUG time [s]	9.21 (8.58–11.20)	9.43 (8.31–10.30)	9.60 (8.50–12.30)	0.235	− 0.013
	Gait speed [m/s]	1.12 (1.09–1.38)	1.06 (0.99–1.26)	1.01 (0.88–1.10)	**0.013** ^**a**^	0.118
	Step length [cm]	58.5 (54.3–61.0)	54.5 (52.0–58.8)	55.0 (48.5–56.0)	**0.032** ^**a**^	0.085
	Double support [%]	18.6 (15.5–20.3)	19.4 (17.5–21.2)	19.9 (18.1–21.9)	0.173	0.027
	Cadence [steps/min]	106 (102–110)	105 (100–109)	103 (98–106)	0.273	0.011
Dual-task	TUG time [s]	9.45 (8.41–9.80)	9.99 (8.21–10.43)	11.00 (9.87–12.66) ^+^	**0.001** ^**a**^	0.207
	Gait speed [m/s]	1.09 (1.03–1.18)	1.00 (0.92–1.18) ^+^	0.88 (0.76–0.98) ^+^	**<0.001** ^**a, b**^	0.278
	Step length [cm]	56.5 (54.3–60.8)	54.0 (50.6–59.3)	51.0 (47.3–56.0)	**0.014** ^**a**^	0.116
	Double support [%]	18.7 (16.6–20.4)	19.9 (18.0–21.8)	21.6 (19.5–23.7)	**0.008** ^**a**^	0.135
	Cadence [steps/min]	104 (100–111)	100 (96–106) ^+^	93 (86–100) ^+^	**<0.001** ^**a, b**^	0.232
Fast	TUG time [s]	5.07 (4.67–5.62) ^+^	6.00 (5.35–6.67) ^+^	6.47 (6.07–7.18) ^+^	**<0.001** ^**a, c**^	0.434
	Gait speed [m/s]	1.91 (1.71–2.02) ^+^	1.59 (1.49–1.72) ^+^	1.49 (1.34–1.58) ^+^	**<0.001** ^**a, c**^	0.405
	Step length [cm]	70.5 (68.0 − 77.8) ^+^	67.5 (62.3–72.3) ^+^	63.0 (57.2–66.8) ^+^	**0.013** ^**a**^	0.247
	Double support [%]	13.2 (11.9–14.3) ^+^	14.9 (13.8–16.9) ^+^	16.8 (15.1–18.4) ^+^	**<0.001** ^**a, c**^	0.412
	Cadence [steps/min]	143 (132–147) ^+^	130 (120–141) ^+^	132 (121–139) ^+^	**0.012** ^**a, c**^	0.119

Bonferroni corrected post-hoc test reveals ^a^ significant difference between young and old aged, ^b^ significant difference between middle and old aged, ^c^ significant difference between young and middle aged; ^+^ significant difference to preferred condition (Wilcoxon test); TUG, Timed Up and Go test; IQR, interquartile range

#### Dual-task condition

**Fig. 2:**
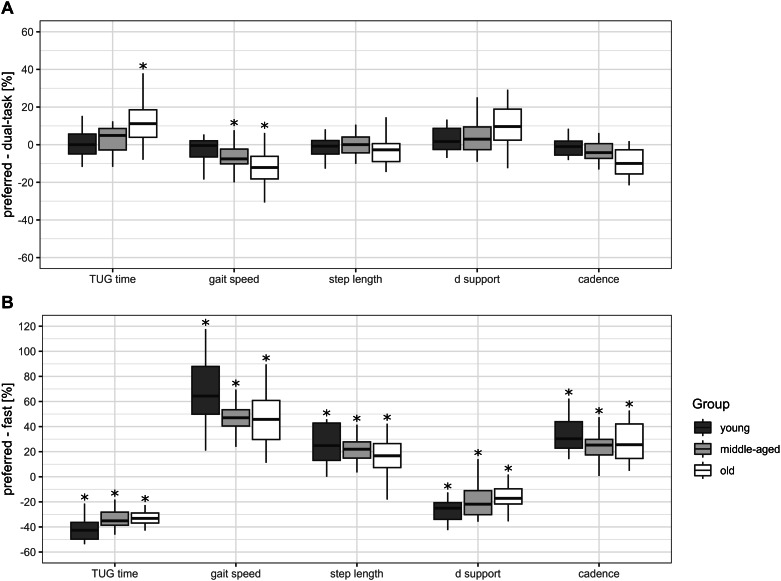
Percentage change in dual-task and fast TUG performance in relation to the preferred condition. Figure 2 shows the percentage change (y-axis) of TUG- and gait-related performance parameters (x-axis) from preferred condition to **(A)** dual-task and to **(B)** fast condition in the three groups (young, middle-aged and old). Box-and-whisker plots showing the lower quartile (25th percentile), median (50th percentile), upper quartile (75th percentile), and degree of dispersion as 95% confidence interval (95% CI). Significant differences in relation to the preferred condition (zero line) are marked (*) with an asterisk and correspond to a significance level of p < 0.05. d support, proportion of double support phase during a step

The dual-task condition revealed a larger group difference than walking at the preferred gait speed (Table 2). All gait parameters differed among groups. Post-hoc analysis showed that the old group differed from the middle-aged group in gait speed (r = 0.34, p = 0.023), and cadence (r = 0.42, p = 0.024). Furthermore, the young and older adults differed in all recorded parameters (TUG time, r = 0.58, p = 0.001; gait speed, r = 0.66, p < 0.001; step length, r = 0.46, p = 0.010; double support, r = 0.49, p = 0.006; cadence, r = 0.60, p < 0.001). Comparing the dual-task with preferred condition (Table 2; Fig. 2A) revealed a significantly slower gait speed (middle-aged: r = 0.60, p = 0.007; old: r = 0.66, p = 0.003) and lower cadence (middle-aged: r = 0.49, p = 0.028; old: r = 0.83, p < 0.001) in the old and middle-aged groups. Furthermore, the old group’s TUG time (r = 0.77, p = 0.001) in the dual-task condition was significantly longer than the preferred condition. The young group revealed no dual-task-related adaptation compared to the preferred condition.

#### Fast condition

As with the dual-task condition, all gait parameters differed among groups during the fast condition (Table 2). Post-hoc analysis showed that the young group differed from the old and middle-aged groups. The young group needed less time to perform the TUG (middle-aged, r = 0.49, p = 0.005; old, r = 0.81, p < 0.001), walked significantly faster (middle-aged, r = 0.48, p = 0.007; old, r = 0.79, p < 0.001), revealed a shorter double support time (middle-aged, r = 0.47, p = 0.009; old, r = 0.79, p < 0.001) and a higher cadence (middle-aged, r = 0.40, p = 0.034; old, r = 0.41, p = 0.028) than the middle-aged and old groups. The young participants also took significantly longer steps (r = 0.63, p < 0.001) than the old participants. Comparing the fast to the preferred condition, all parameters in all three groups changed significantly in terms of performing faster (p ≤ 0.001) with the young group showing the greatest adaptation (Table 2; Fig. 2B).

#### Arm-movement test

We observed significant group differences during the arm-movement test in the total number of contacts on the platforms and maximum movement speed, while the level of movement fatigue did not differ among groups. Post-hoc analysis revealed that the young participants’ arm-movements differed significantly from those of the old (number of contacts: r = 0.63, p < 0.001, max. speed: r = 0.71, p < 0.001) and middle-aged participants (number of contacts: r = 0.40, p = 0.032, max. speed: r = 0.45, p = 0.015; Table 3).

**Table 3:** Performance of repetitive goal-directed arm-movement test of young, middle-aged and older adults

Parameter	Young (18 – 34 years) median (IQR)	Middle-aged (35 – 59 years) median (IQR)	Old (60+ years) median (IQR)	p-value overall	η²
	n=20	n=20	n=20	n=60	n=60
Number of contacts [N]	97.8 (92.1 – 114.9)	86.9 (77.0 – 112.9)	85.6 (75.6 – 98.0)	<0.001 ^a, c^	0.250
Maximum speed [m/s]	3.2 (2.9 – 3.9)	2.7 (2.3 – 3.7)	2.5 (2.2 – 3.0)	<0.001 ^a, c^	0.328
Fatigue [%]	0.8 (0.8 – 1.1)	0.9 (0.8 – 1.1)	0.9 (0.8 – 1.1)	0.471	-0.009

Bonferroni corrected post-hoc test reveals ^a^ significant difference between young and old aged; ^b^ significant difference between middle and old aged; ^c^ significant difference between young and middle aged; IQR, interquartile range

### Correlations between the fast TUG and arm-movement test

**Fig. 3:**
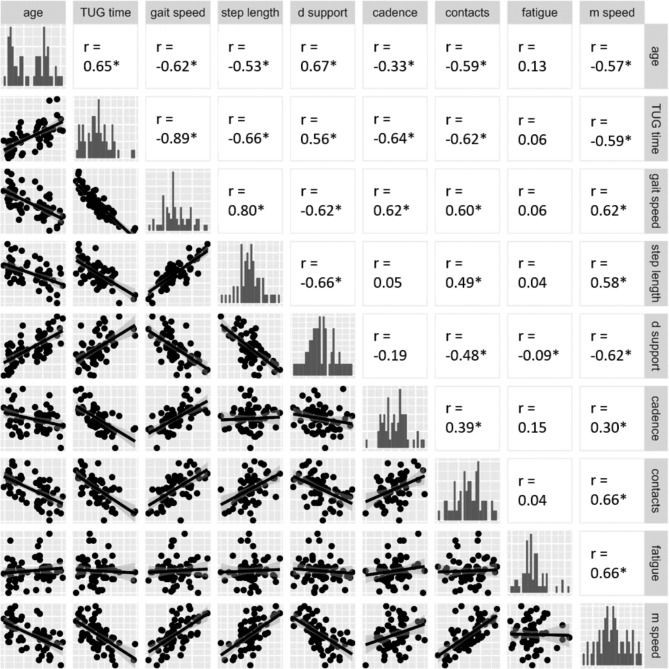
Correlations between age, fast TUG- and arm-movement The figure shows scatter plots with regression lines for correlations of age, fast TUG time, gait-related parameters of fast TUG (gait speed, step length, double support proportion, cadence) and arm-movement (number of contacts, fatigue, maximum arm-movement speed) in the lower left section. The diagonal line shows a histogram of each parameter. The upper section shows results of the Pearson correlation D support, proportion of double support phase during a step; contacts, number of contacts; m speed, maximum arm-movement speed; * p < .05

Our correlation analysis revealed significant correlations between the TUG test parameters during the fast condition (_fast_) and arm-movement test: TUG time_fast_ and gait speed_fast_ strongly and step length_fast_, double support_fast_, and cadence_fast_ correlated moderately with the number of contacts and maximum arm-movement speed (Fig. 3). Fatigue yielded no correlations. All parameters except for fatigue correlated strongly with age.

**Fig. 4:**
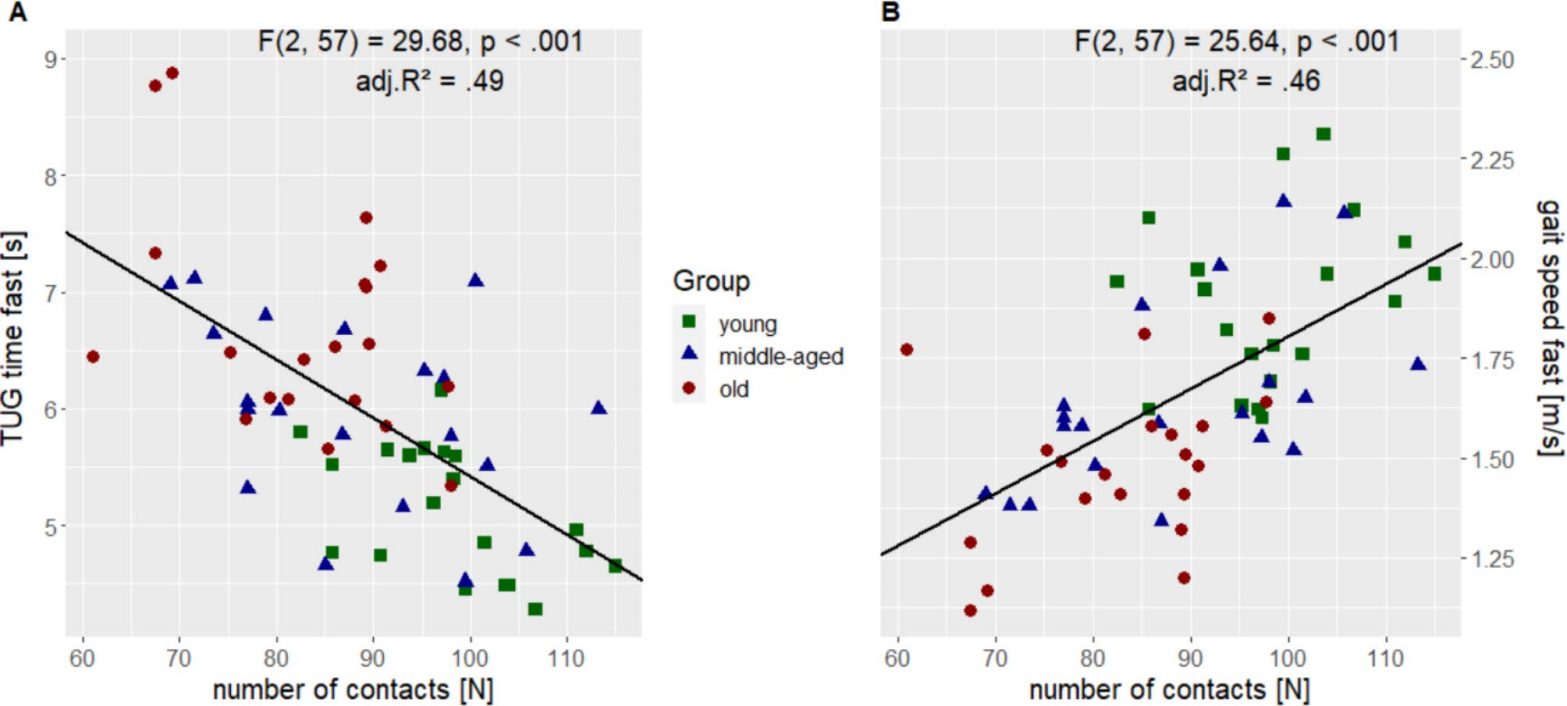
Visualization of the multiple regression analyses The scatter plot shows the relationship between the number of contacts (x-axis) and **(A)** TUG time (left y-axis) or **(B)** gait speed (right y-axis) of the fast TUG condition, respectively, across all groups (group allocation is marked by different color and shape). Based on the applied model, the results of the multiple regression adjusted for age are placed in the top right corner (gray box) of the corresponding figure part

Relying on our correlation results, we calculated two multiple linear regression models with TUG time_fast_ and gait speed_fast_ as dependent variables and the number of contacts and age as explanatory (independent) variables. We noted a significant regression equation for TUG time_fast_ (F(2, 57) = 29.68, p < 0.001) and gait speed_fast_ (F(2, 57) = 25.64, p < 0.001). 49% of the variance of TUG time_fast_ (adjusted R² = 0.49) and 46% of variance of gait speed_fast_ (adjusted R² = 0.46) can be explained by the number of contacts and age parameters (Fig. 4). Both age (estimated as 0.024 s; p < .001, partial r = 0.449) and number of contacts (estimated as -0.029 s; p = 0.002, partial r = -0.388) qualified as a significant predictors for TUG time_fast_. For gait speed_fast_, age (estimated as -0.006 m/s; p < 0.001, partial r = -0.418) was a predictor, as were the number of contacts (estimated as 0.008 m/s; p = 0.004, partial r = 0.370). The estimated reduction in TUG time_fast_ equalled − 0.029s per contact (0.3s per 10 contacts) and the estimated increase in gait speed_fast_ equalled 0.008 m/s per contact (8 cm/s per 10 contacts).

## Discussion

The main aim of this study was to test our hypothesis that the decline in TUG performance and arm-movements (repetitive goal-directed) with age reflect the same motor degradation. To determine which TUG condition is best suited for this experimental purpose, we tested three different conditions: preferred speed, fast speed, and dual-task. Comparing the TUG’s walking sequence in three different age groups revealed that the TUG condition influenced group differences: The young group was able to improve their TUG performance from preferred to fast speed the most, while the dual-task impaired the old group’s TUG performance. We also detected strong correlations between the performance of repetitive goal-directed arm-movements and fast TUG performance.

Our study investigated the relationship between upper-limb performance and gait parameters, specifically in the context of repetitive goal-directed arm-movements and the TUG test. We observed significant correlations between the number of contacts in the arm-movement test and gait parameters in the fast TUG condition. We also noted that arm-movements (number of contacts and maximum speed) were similarly affected by age as in the TUG performance. Little has been known until now about the relationship between multi-joint upper-limb movements and walking, or specifically the TUG performance. Upper-limb performance as finger tapping, and diadochokinetic movements have been extensively studied in diverse patient groups, but without considering the connection to walking abilities [31–37]. Some research results suggest that changes in finger-tapping rates are related to central motor conduction times and are therefore suitable to track performance status, e.g., in multiple sclerosis. Additionally, finger tapping could serve as a useful tool to assess cognitive status in different contexts, e.g., Parkinson disease or multiple sclerosis, when assessing disease progression [31, 36–38]. Interestingly, Hausdorff et al. postulated that walking might be more closely related to complex motor tasks than to isolated finger tapping [32]. This may be why less complex upper-limb tests in healthy subjects have failed to prove any interrelation to walking [32, 35, 39]. Our arm-movement test implies a multi-joint movement from the synergistic interaction between shoulder, elbow, and wrist for successful execution. Walking also involves multiple joints and requires complex control mechanisms to generate propulsion while maintaining posture [40]. Studies focusing on locomotor-like movements evoked by rhythmic arm movements reported functional neural coupling between upper and lower limbs [41–44]. It is assumed that rhythmic arm and leg movements share a common neural control [44–46]. Based on our findings, we also suggest that the fast executions of TUG and arm-movement test may challenge the motor system by making similar demands. Thus, walking and arm-movement parameters may reflect two facets of the same motor degradation of coordinated rhythmic movements [45, 47, 48]. This might be surprising given the fact that walking, another than arm movements, comprises equilibrium capacity. By using multiple regression analysis, we found that the parameters number of contacts and age have predictive value on TUG time and gait speed in the fast TUG condition. A person achieving a high number of contacts seems to walk faster, leading to a shorter total TUG time. These dependencies are also age-related, that is, the upper-limb performance of older subjects is reduced, similar to the decrement in TUG performance. We suggest estimating relevant changes in increments of 10 contacts (e.g., 10 more contacts mean a 0.3s slower TUG time or 8 cm/s slower gait speed, respectively, and 0.2s slower TUG time per age decade or a 6 cm/s slower gait speed). Since gait speed and TUG time are reliable predictors for overall health and mobility [4, 18, 49], we maintain that repetitive, goal-directed arm movements have the potential to serve as a health-related predictor as well. We assume that our correlation findings might apply to patients unable to walk safely (e.g. TUG test or walking sequences) [35]. Incorporating upper-limb movement tasks within biomechanical models of motor behavior may raise their clarifying value.

A wide overall range of reference values for TUG duration has been reported. Its broad distribution among diverse age groups implies variations in TUG execution and instruction [50, 51]. In other words, the significance of TUG results could probably be enhanced by specifying instructions based on specific study objectives. Furthermore, we assume that analyzing TUG and especially its sequences via motion capture systems will considerably improve the explanatory power of TUG - an unquestionably reliable instrument for assessing mobility [16]. TUG is usually performed at subjects’ preferred movement speed. This condition revealed no significant differences in TUG time among our groups. Interestingly, when analyzing TUG more closely, e.g., by extracting the walking sequence of TUG at preferred movement speed, significant differences appeared in gait speed and stride length between the young and old groups. There is ample evidence of reduced gait speed and stride length with age [52, 53]. In general, the natural aging process implies a continuous decline in muscle strength [54] that can alter joint kinetics and kinematics in terms of a distal-to-proximal (ankle to hip) shift of joint work [55–57]. This alteration affects the body’s propulsion and may result in a shorter stride length [55–57] and thus slower gait.

Adding a cognitive task while performing TUG led to prolonged TUG time in the old group and reduced gait speed and cadence in the old and middle-aged groups compared to the preferred condition. Decelerated movement attributable to a dual-task is a well-studied phenomenon [19, 58–60]. Executing a cognitive task while walking, more specifically while doing TUG may interfere with the cognitive control that coordinated movements require [3, 19, 61] resulting in a slower movement speed. The strength of the dual-task’s effect depends on its complexity level for each individual [19, 62]. From the physiological perspective, executing the two tasks simultaneously and correctly requires an increase in cortical activation. Aging generally implies a breakdown in network activity and connectivity between cortical regions involved in the conception, initiation, and on-going control of motor and cognitive processes [63]. With age, there is also a shift from activity in the brain’s posterior regions to increased activity in the prefrontal cortex, which is involved in executive functions (attention and working memory) [64]. However, the prefrontal cortex has limited capacity, and overreliance on this region for compensatory purposes may detract from other cognitive processes such as multitasking [64, 65]. These structural changes caused by aging lead to inter alia inappropriate allocation of attentional resources and impaired executive function [54, 63] associated with postural instability [3, 66, 67]. The present study may reflect this aging effect through the growing influence of dual-task on TUG performance as we age. This means that the concurrent execution of required motor and cognitive tasks resulted in our old group’s a clearer delineation from the young and middle-aged groups, unlike in the preferred condition. The older group differed significantly from the young group in TUG time and all gait parameters, and from the middle-aged group in gait speed and cadence. Accordingly, our study’s older participants reported more falls than the younger ones did. We assume that our old group’s clear differentiation by provoking cognitive-motor interference emphasizes the importance of analyzing motor behavior under various conditions to identify specific risk constellations.

Our individual subjects’ performance during the fast TUG condition revealed their capacity to voluntarily increase movement speed, which is considered an indication of overall vitality [4, 5, 18, 68, 69]. All age groups increased their movement speed significantly compared to the preferred condition. This increase is related to adapting temporal, spatial, and rhythmic aspects of gait, in line with the recent literature [70, 71]. Even if our older participants exhibited smaller effects from the preferred to the fast condition, all groups seem to follow the same strategy, i.e., reduced double support time, longer step length and enhanced cadence. The age-related continuous decline in neuromuscular performance also means a lower force production rate restricting the body’s propulsion [72, 73]. Age’s impact on moving fast strengthens the differences between groups in the fast TUG condition and the arm-movement test, which is also a fast movement task. It is common knowledge that aging first affects our fast twitch muscle fibers essential for explosive force [74, 75] leading, e.g., to a greater drop in maximum gait speed compared to a comfortable gait speed over decades as an adult ages [76, 77]. Thus, in contrast to the preferred TUG condition, both fast conditions reveal significant group differences, and they especially help to distinguish the young group from the middle-aged and old groups – unlike the dual-task condition, where old age was the distinguishing factor. We conclude that the fast conditions address another aspect of aging that becomes evident earlier in the life span than does the challenge of cognitive-motor interference.

## Limitations and future perspectives

Analyzing TUG yields multidimensional information about motor behavior in everyday demands. Since we extracted the TUG walking sequence to analyze gait, we accepted a relatively short distance, which obviously implies acceleration and deceleration phases. This fact limits comparison with reference values recorded during continuous walking over a longer distance. However, we are convinced of TUG’s value, as it condenses various everyday tasks relevant for autonomy and thus reveals functional status. As TUG can deliver much more information than illustrated here, future investigations will need to focus on the sit-to-stand, turning, and stand-to-sit sequences. Moreover, we have not exhausted all the potential of capturing whole-body motion. In future analyses, we hope to derive additional movement parameters, e.g., from joint velocities during TUG and arm-movement.

## Conclusions

In the present study, we reproduced the finding that TUG performance as well as repetitive goal-directed arm-movements depend strongly on age. We have shown that age-related impairments in TUG performance are intensified when performing the TUG fast or during a dual-task. Interestingly, we were able to demonstrate that the TUG performance declines with age, especially in fast execution, and that that factor is closely related to the performance of repetitive goal-directed arm-movements. We assume that walking fast with fast, repetitive, goal-directed leg-movements and fast repetitive goal-directed arm-movements reflects two facets of the same motor degradation in coordinated repetitive movements, which might be even more relevant to fast walking than are equilibrium capacities. Accordingly, this interrelation may have significant relevance regarding the functional assessment of patients unable to walk safely.

## Data Availability

This article only includes summarized data from this study. Raw data is available from the corresponding author upon reasonable request.

## Abbreviations

TUG: Timed Up and Go test
TUG_fast_: TUG test at fast condition
Arm-movement test: fast repetitive goal-directed arm-movement test
POMA: Performance Oriented Mobility Assessment
FES-I: Falls Efficacy Scale – International
IQR: Interquartile range
